# A First-Hand Picture of the Kaiser and Berlin in War Time

**Published:** 1918-08

**Authors:** Walter W. Sage


### A FIRST-HAND PICTURE OF THE KAISER AND BERLIN IN WAR TIME

BY WALTER W. SAGE, D.D.S.

Since the war started few close-up views of the Kaiser have been presented by the horde of writers taking the world war as a theme. This is due, primarily, to the fact that few people ever get in touch with the Emperor when he is relaxed. Those who do come in contact with him have little to say, they being chiefly members of his household, military advisers and attendants.

I treated the Emperor several times. He usually came to my associate's office in Berlin, but the last time I saw him I was summoned to the palace at Potsdam. One of the many secretaries the Emperor has at his disposal called up and said he would like to have me attend the Kaiser at ten o'clock the following morning. So early on the appointment day I packed my instruments and, with a foot-power dental engine under my arm, took a train for Potsdam. At the Potsdam station a royal carriage met me and I swung out to the palace in style. I was shown to a large room on the second floor of the imperial quarters. It must have been a wardrobe of some sort, because there were uniforms of all sorts covering the walls. A heavy chair with a reclining back was placed at my disposal to serve as a dentist's chair. After arranging my instruments I stepped into an ante-chamber to await the command of whoever had his eye upon me.

Promptly at ten o'clock—the Kaiser is always on the dot in keeping appointments—a functionary informed me that the Emperor awaited my coming. As I crossed the threshold, the Kaiser saw me. He shifted his head on the chair back and said: "Hello, there, Sage." The Kaiser speaks perfect English, far better than what we hear, as a rule, in this country.

As far as I could observe, there were no signs of gloom or a bent back which might be traced to the effects of the European war. The Kaiser has grown quite gray during the last few years, but he is very active, moves around with snap, and is what Americans would call "a live wire." He joked about the high price of the American dollar measured in marks.

"Well, I see that the dollar is going up every day. That must make Americans happy," the Kaiser said with a smile. He also referred to the progress which German dentists are making, and seemed to think they would soon equal the skill which Americans have shown in this profession.

At one stage of the treatment the Empress came into the room to see how things were getting along. She inquired, in English, if the pain was unbearable, and the Kaiser said it was pretty bad, but he guessed he could stand it.

Perhaps you would like to know the cause of my visit. Well, the Kaiser said he was over in France eating grouse when he bit a piece of shot which was imbedded in the meat served him. The shot broke off a piece of tooth, and he wanted me to put another piece on for him. He smiled at the thought of biting a piece of shot.

In regard to the present conditions in Berlin, all the food, clothing and other necessities are regulated in respect to sale by the police department, which is today just as efficient and well set up as it was before the war. Practically everything is sold according to the card system. The cards are, in reality, a series of coupons which call for so much a week. If you use all your coupons on the first day of the week you don't get any more until the week is up. This, of course, absolutely regulates supply and prices. The police issue the cards and collect the coupons and check up the whole business of Berlin every day.

When I left Berlin, sixty grammes of butter was a week's allotment. That means just enough butter to spread one roll each day in the week. Soap was sold at the rate of one cake a month for an inhabitant. Two eggs were supposed to last three weeks. If they didn't you were refused more until the expiration of that time. There is absolutely no lard in the country, and fats, such as bacon and pork, are very scarce. Sorghum is served with coffee at even the leading hotels. There are now two days in the week on which the eating of meat is forbidden. A man has to prove that he needs a suit of clothes very badly before he is allowed to buy one. Awhile ago the Government found that a department store was overcharging its customers, and the police closed it up.

Getting across the German frontier was a tedious task. You must go to the police and get photographed, for one thing, and four prints are made from the plate. One of them is sent to the authorities at the place you designated as the one where you wish to pass out of the country. The police attend to only forty cases a day, and as the demand for passes is great you must get up very early in the morning if you expect to get accommodated. I made four visits to the precinct headquarters before my case was passed upon. I went to the station at six thirty o'clock on the morning of the fourth day and got a number.

When you arrive at the frontier the photograph you carry is compared with the one forwarded to the border by the police. If the pictures check up you are allowed to pass, after much signing of names and data gathering. Such formality applies not only to one who is going to sail for America. It applies to every one who seeks to cross the frontier in either direction. As a consequence, Germany knows every minute of the day exactly where she stands in regard to men, women and children, as well as in regard to supplies for meeting their wants.

Wounded soldiers are just beginning to be seen on the streets of Berlin, although the war is now well into its third year. Soldiers preparing for the front march up and down the streets of the capital every little while. They range in age from seventeen to forty-five. When the war started, conversations over the telephone were limited to the German language. Lately this rule has been slackened, and English can be used without central shutting you off.

Although women are running the street cars and digging the new subways, there does not seem to be a scarcity of men in the city. The orchestras, numerous there as before the war, all seem to have their full quota of men. Theaters, restaurants and the big hotels are running full blast, and a general air of merriment prevails. The Government asked some time ago that mourning garb be dispensed with, and little of it is seen. Dancing is about the only amusement which is not permitted.—*Dental Forum.*

